# Neuroprotective potential of selenium nanoparticles and/or physical and mental activities against social isolation–induced depression in a rat model: inflammatory, oxidative stress, apoptotic, and neurotransmission modulatory pathways

**DOI:** 10.3389/fphar.2026.1810142

**Published:** 2026-07-14

**Authors:** Karema Abu-Elfotuh, Furqan Hashim Hussein, Roaa Hameed Alwaidh, Qutaiba A. Qasim, Layla A. Al-Kharashi, Fatma M. Mostafa, Enji Reda, Abdou Mohammed Ahmed Elsharkawy, Heba Abdelnaser Aboelsoud, Shaimaa R. Abdelmohsen, Magy R. Kozman, Nervana Mostafa Kamal Bayoumy, Yasmen F. Mahran, Abeer Altamimi, Khaled Ragab Abdelhakim, Ahmed M. E. Hamdan, Aliaa Osman Abou-Beih

**Affiliations:** 1 Clinical Pharmacy Department, Faculty of Pharmacy (Girls), Al-Azhar University, Cairo, Egypt; 2 Department of Pharmacology and Toxicology, College of Pharmacy, Al-Ayen Iraqi University, AUIQ, An Nasiriyah, Iraq; 3 Collage of Dentistry, University of Alkafeel, Kufa, Iraq; 4 Department of Pathology and Forensic Medicine, Faculty of Medicine, University of Kufa, Kufa, Iraq; 5 Department of Clinical Laboratory Sciences, College of Pharmacy, University of Basrah, Basrah, Iraq; 6 Department of Pharmacology and Toxicology, College of Pharmacy, King Saud University, Riyadh, Saudi Arabia; 7 Microbiology Department, College of Pharmacy, Al-Ayen Iraqi University, AUIQ, An Nasiriyah, Iraq; 8 Department of Pharmacology & Toxicology, Faculty of Pharmacy, Sinai University – Kantara Branch, Ismailia, Egypt; 9 Anatomy Department, Faculty of Medicine, Al-Azhar University, Cairo, Egypt; 10 Department of Basic Medical Science, College of Medicine, Prince Sattam Bin Abdulaziz University, Al-Kharj, Saudi Arabia; 11 Anatomy and Embryology Department, Faculty of Medicine for Girls, Al-Azhar University, Cairo, Egypt; 12 Clinical Pharmacy Department, Faculty of Pharmacy, Misr University for Science and Technology, Giza, Egypt; 13 Physiology Department, College of Medicine, King Saud University, Riyadh, Saudi Arabia; 14 Department of Pharmacology and Toxicology, Faculty of Pharmacy, Ain Shams University, Cairo, Egypt; 15 Natural and Health Sciences Research Center, Princess Nourah bint Abdulrahman University, Riyadh, Saudi Arabia; 16 Department of Histology, Misr University for Science and Technology, Giza, Egypt; 17 Department of Pharmacy Practice, Faculty of Pharmacy, University of Tabuk, Tabuk, Saudi Arabia; 18 Faculty of Pharmacy in Prince Fahad Bin Sultan Chair for Biomedical Research (PFSCBR), Tabuk, Saudi Arabia; 19 Department of Clinical Pharmacy, Ain Shams University Specialized Hospital, Cairo, Egypt; 20 Department of Pharmacy Practice, Sinai University, Kantara, Egypt

**Keywords:** depression, mental activity, physical activity, selenium nanoparticles, social isolation

## Abstract

**Background:**

Social isolation (SI), attributable to modern lifestyles and fast-growing technology, is a leading cause of depression. Selenium nanoparticles (Se-NPs) are neuroactive agents owing to their antioxidant and anti-inflammatory activities. Additionally, physical and mental activities (Ph&M) exert neuroprotective effects by optimizing the release of both neurotransmitters and growth factors. However, their neuroprotective effects against SI-induced depression are still poorly investigated.

**Aim:**

We aim to explore the neuroprotective effect of Se-NPs, Ph&M, and their combination to guard against the harmful effects of SI-induced depression in a rat model.

**Methods:**

Fifty Sprague Dawley rats were randomly allocated into five groups: control, SI, Ph&M, orally administered Se-NPs (0.1 mg/kg), and a combination group. Neuroprotective activity was quantitatively estimated pharmacologically, biochemically, histologically, and behaviorally.

**Results:**

SI caused behavioral and biochemical alteration in the rat model, decreasing neurotransmitter levels, increasing the transcription of inflammatory response genes (*TLR4* and *NF-κB*), and consequently increasing the production of the cytokines TNF-α and IL-1β. It also activated the proinflammatory NLRP3/caspase-1 pathway. The ER stress parameters PERK, CHOP, and GRP78 were significantly elevated. Impairment of autophagy and increased neurodegeneration were detected via the decline of AMPK/SIRT-1/Beclin-1 PI3K/AKT gene expression and m-TOR overexpression. Decreased expression of TrkB and CREB mRNA and consequent decline in brain-derived neurotropic factor were recorded. SI caused a drastic drop in Wnt3a and β-catenin levels and increased GSK3β activity affecting neuroplasticity and cognitive functions. Administration of Se-NPs and/or application of Ph&M, especially their combination, provided significant protection against prior SI effects.

**Conclusion:**

Se-NPs and Ph&M, especially their combination, showed promising protective effects against neuroinflammation, oxidative stress, apoptosis and subsequent alterations of test animal behaviors precipitated by SI.

## Highlights

- Social isolation (SI) induces depression by initiating alteration of neurotransmitters and growth factor release, in addition to creating neuroinflammation, oxidative stress, and apoptosis.
- Administration of selenium in nanoparticle form (Se-NPs) guards against SI-induced depression.
- Practicing physical and mental activities (Ph&M) protects against SI-induced depression as well.
- Regulation of Wnt3/β-catenin/GSK3β, AMPK/SIRT-1/Beclin-1, CREB/BDNF/TrkB, and PI3K/AKT/m-TOR signaling pathways hinders depression-like symptoms precipitated by SI.

## 1 Introduction

Depression is the most common serious mental mood disorder characterized by complex emotional and cognitive behaviors. It has different grades with various opposite subjective symptoms. Its etiology includes genetic, neurological, hormonal, immunological, and neuroendocrinological factors that center on reactions to stressors (Burback et al., 2024). For example, stressful life events, loneliness, and social isolation (SI) (Abu-Elfotuh et al., 2022a) induce depression-like symptoms that resist treatment with many traditional antidepressive agents. Treatment options for depression include pharmacologic therapy, psychological support, and lifestyle modification. Meanwhile, many patients experience treatment resistance due to varied factors, including genetic factors (Fabbri, 2025). Therefore, a combination of pharmacologic and non-pharmacologic approaches can help achieve the best therapeutic outcome against symptoms of depression (Taheri Zadeh et al., 2021).

Stress is an adverse stimuli that negatively impacts body homeostasis, with resultant physiological and psychological responses (RAMEZ et al., 2019). Stress is an environmental risk factor for the incidence of psychiatric disorders (Varese et al., 2012). Physiological responses to stress occur via activation of the sympatho–adrenomedullary (SAM) and hypothalamic–pituitary–adrenocortical (HPA) axes. The SAM axis comprises the immediate response to stress with the release of epinephrine and norepinephrine (NE) from the adrenal medulla and NE from the sympathetic nerves. The HPA axis comprises a prolonged response to stress, which eventually results in glucocorticoid secretion from the adrenal cortex. This leads to sustained elevation in the glucocorticoid level, which induces structural and functional changes in the brain, specifically the prefrontal cortex and hippocampus, which regulate behavior and emotions and process psychogenic stress (Elfakharany et al., 2024). Hence, chronic severe stress can upregulate the expression of pro-apoptotic proteins, such as caspase-3. In contrast, the release of neurotransmitters that regulate mood and behavior, such as serotonin is greatly diminished in both human and animal models (Puglisi-Allegra and Andolina, 2015). Chronic mild stress similarly induces a depressive-like behavior associated with increased proinflammatory microglial activation as seen in the study conducted on rats by Habib et al. (2020). It is evidenced that neuronal oxidative stress develops because of psychological stress, with consequent DNA damage, lipid peroxidation and alterations in protein functioning, as seen in multiple studies on gerbils, mice and rats (Calcia et al., 2016). The higher susceptibility of the brain to reactive oxygen–species mediated damage could be attributed to several factors (Has et al., 2022), including its high oxygen consumption, high lipid content, and low antioxidant levels.

SI in rodents is a common animal model used to study different facets of mental disorders, as it induces long-lasting alterations in functional connectivity, behavior, and molecular expression (Reinwald et al., 2018). SI is a powerful stressor for both rodents and humans. In humans, SI is associated with a higher risk of mental health problems, such as depression and anxiety, in addition to the increased risk of mortality. In rodents, SI induces anxiety and Depression-like behaviors, aggression, and memory impairment (Abu-Elfotuh et al., 2022a).

The objective of the current study is to assess the protective effects of selenium (Se) and physical and mental activities (Ph&M) against SI-induced depression in an attempt to minimize reliance on mainstay pharmacologic therapies, mainly antidepressants, which may carry devastating adverse effects themselves. If Se and Ph&M are proven to be successful, they can be recommended for protection against SI-induced depression.

Se is a trace element that is essential for mental health and brain function (Ferrei et al., 2021; Sajjadi et al., 2022). It has been proved that Se has neuroprotective properties (Rayman, 2012).

In the current study, we explore the neuroprotective role of Se nanoparticles (Se-NPs) that have higher cellular entrapment after oral administration due to their high bioavailability and cellular endocytosis. Se-NPs also exhibit higher antioxidant activity than other physical or chemical forms of Se. Furthermore, Se-NPs have less toxicity than Se (Elfakharany et al., 2024).

To obtain a clear view of the impact of SI and the suggested protective therapies a variety of pathways and genes are assessed in the current study.

Wnt3a, β-catenin, and GSK3β are key regulators of synaptic plasticity and neurogenesis, known to be dysregulated in chronic stress and targeted by antidepressant therapies (Liu et al., 2019). The central mediators of neuroinflammation are NF-κB, TNF-α, and IL-1β, which are hallmarks of SI-induced depression (Habib et al., 2020). Another biomarker of neuroinflammation and microglial activation recently linked to stress-induced neuropathology is CHI3L1. In addition, the brain-derived neurotropic factor (BDNF) is a critical neurotrophin for neuronal survival and synaptic plasticity, whose expression is enhanced by both physical activity and antidepressants (Bai et al., 2019).

The genes selected for RT-qPCR analysis were chosen to comprehensively assess the key molecular pathways implicated in depression and the potential protective effects of Se-NPs and Ph&M. These include apoptosis (Bax and Bcl-2) for evaluating the balance of cell survival or death and autophagy (Beclin-1, AMPK, SIRT-1, and m-TOR) for assessing the regulation of cellular clearance mechanisms, which are impaired in stress-related disorders. To quantify the activation of the innate immune response, inflammasome and inflammation (NLRP3, caspase-1, NF-κB, and TLR4) are to be measured. For ER stress, CHOP, GRP78, and PERK are chosen to evaluate the unfolded protein response, a key mediator of chronic stress-induced neuronal damage. Neurotrophic signaling (CREB and TrkB) are chosen to assess the capacity for synaptic plasticity and neuronal resilience. Antioxidant defense (Nrf2 and HO-1) are chosen to evaluate the activation of the endogenous antioxidant system, a known target of Se.

## 2 Methodology

### 2.1 Drugs and chemicals

Se-NPs (CAS Number: 7782-49-2)—150-nm particle size (product number: 919527)—were purchased from Sigma-Aldrich Co. (St. Louis, MO, United States) and dispersed in aqueous medium to produce 0.15 wt% by sonication. Their shape and size were examined using transmission electron microscopy at 200 kV (JEM-2100F; JEOL, Tokyo, Japan). In addition, their fingerprint or composition was measured using Fourier transform infrared spectroscopy in the wavenumber range of 4,000–400 cm^−1^.

### 2.2 Animals

Fifty 18-week-old healthy male Sprague Dawley rats, with body weights between 250 and 280 g, were procured from the Nile Company for Pharmaceuticals and Chemical Industries in Cairo, Egypt. The animals were kept in stainless-steel cages under controlled environmental conditions, with an ambient temperature of 25 °C ± 1 °C and a 12-h light–dark cycle (lights on at 7 a.m.). For 5 weeks, the isolated rats were separately housed in cages shielded with black plastic, whereas the socially housed rats were randomly paired and kept in cages without covers. All animal handling and experimental procedures adhered to the Guidelines for the Care and Use of Laboratory Animals published by the National Institutes of Health (NIH Publication No. 8023, revised 1978). The study is reported in compliance with the ARRIVE guidelines. The study protocol was approved by the Animal Care and Use Committee of the Faculty of Pharmacy, Al-Azhar University (ethical approval number 515/2025).

### 2.3 Design of the experiment

Fifty rats were randomly divided equally into five groups of 10 rats each. The rats were categorized in the following manner:

Control socialized group: Rats were kept in clear, covered cages, given distilled water (1 mL/kg), and paired randomly.

Socially isolated (SI) group: Rats were kept separately (one rat per cage) in cages covered with black plastic and given distilled water (1 mL/kg) (Yavuz et al., 2024).

SI group protected by Ph&M: The isolated rats received 1 mL/kg of distilled water and were subjected to the Ph&M protocol through an open field test (OFT) and a swimming test twice a week for 5 weeks (Arslankiran et al., 2025).

SI group protected by Se-NPs: The isolated rats were given Se-NPs (0.1 mg/kg) orally (Elfakharany et al., 2024).

SI group protected by both Se-NPs and Ph&M (COMB): The isolated rats were given Se-NPs (0.1 mg/kg) orally and were subjected to Ph&M protocol through an OFT and a swimming test twice a week for 5 weeks (Arslankiran et al., 2025). Se-NPs (0.1 mg/kg) was given orally during these 5 weeks (Elfakharany et al., 2024).

At the end of the experiment, the six animals were subjected to the Y-maze task (Magnuson et al., 2007), forced swimming test (FST), and OFT (Yankelevitch-Yahav et al., 2015). Subsequently, the animals were euthanized, and their brains were promptly removed and then rinsed with an ice-cold saline solution. Four tissue samples from each group were fixed in 10% neutral buffered formalin for histopathological examination. The remaining brain tissue in each group was dissected into two parts. The first portion of the brain tissue was homogenized in ice-cold Tris-HCl 50 mM (pH 7.4) (10% w/v) supplemented with 300 mM sucrose to attain 10% homogenate (w/v), which was then centrifuged at 1800 *g* for 10 min at 4 °C to obtain the supernatant for biochemical measurements. The second share of the brain tissue was rapidly frozen at −80 °C until they were used for real-time PCR (RT-PCR) analyses.

### 2.4 Physical & Mental activities (Ph&M) Protocol

Ph&M was used as a protective tactic against SI-induced depression. Ph&M was performed twice weekly by subjecting animals to FST and Y-maze on two days per week for 5 weeks.

### 2.5 Behavioral studies

#### 2.5.1 Forced swimming test

This test is designed for assessing depressive-like behavior as previously detailed (Yankelevitch-Yahav et al., 2015; Shen et al., 2019). On the day before the measurements, individual rats were placed into a cylindrical tank (with a diameter of 40 cm and a height of 50 cm) filled with fresh water (maintained at 24 °C ± 2 °C) to a depth of 30 cm. The rats were allowed to swim for 15 min, after which they were removed from the cylinder, dried, and returned to their original cages. Twenty-four hours following the pretest session, the rats were again placed in the tank for 5 min, and the following behaviors were recorded by an observer who had been previously trained and was blinded to the Se-NPs and/or Ph&M treatments: immobility (the time spent floating immobile or with only small limb movements to keep the head above the water), swimming (the time spent actively swimming), and climbing (the time spent making upward movements of the forepaws directed toward the cylinder wall). These parameters will be recorded as an index of decision-making, muscular strength, neuromuscular coordination, awareness and vigilance, as well as attention and learning ability.

#### 2.5.2 Open field test

OFT was employed to assess changes in rat latency, locomotor activity, and rearing behavior. As previously outlined (Cunha and Masur, 1978; Abu-Elfotuh et al., 2022b), a square wooden box measuring 80 × 80 × 40 cm, featuring red polished walls and a white floor, was utilized. The apparatus was divided into 16 (4 × 4 cm) square units using black lines. Each animal was gently placed in the center of the open field and allowed to move freely. Over a 3-min period, three parameters were carefully observed and recorded: the latency time (the time spent before the animal began to move), the frequency of ambulation (representing the number of squares entered by the rats with all four limbs), and rearing frequency (the number of times the rat stood on its hind paws). These parameters will be recorded as indices of locomotor activity, excitability, emotionality and exploratory behavior in rodents.

#### 2.5.3 Y-maze spontaneous alternation test

Spatial working memory, a form of short-term memory, can be reflected in an animal’s inclination to explore new environments (Hritcu et al., 2012). A black wooden Y-maze with three arms labeled A, B, or C and a symmetrical triangular central region were utilized. Each arm was positioned at 120° from the others. The rats were briefly positioned at the edge of one arm and given 8 min to freely move around the maze. When the rat’s hind paws were fully extended inside the arm, the entries were counted. The following formula was used to determine spontaneous alternation (SAP) based on the total number of arm entries and alternations: SAP (%) = [number of alternations/(the total arm entries − 2)] × 100] (Michaelson, 2014).

#### 2.5.4 Conditioned avoidance response test

Under extremely stressful circumstances, depression in the rats was evaluated using the conditioned avoidance response (CAR) test. As previously described by Li et al. (2020), a special wooden box apparatus with five interconnected chambers and a movable glass was used; four floors were powered by a stimulator set to 50 V and 25 pulses/second, while the fifth chamber’s floor was made of glass (safety area). The rats were trained on the day prior to the experiment. The training consisted of 5 s of auditory stimuli (conditioned stimulus), followed by 5 s of foot shock. For 2 days, the same animals were tested repeatedly. Before administering the electric shock, the number of posttreatment trials (on the first and second days) that each rat needed to reach the safety area within 5 s after the conditioned stimulus was determined (Li et al., 2020).

### 2.6 Tissue sample assembly and preparation

At the end of the behavioral experiments and following behavioral evaluations, the rats were anesthetized with ketamine (80 mg/kg, i.p.). Afterward, the rats were sacrificed by cervical dislocation (Cheng et al., 2020; Abu-Elfotuh et al., 2025). Brain tissue was extracted immediately after being sacrificed and cleaned with cold saline. Four brain tissue samples from each group were stored in 10% formaldehyde and used for histopathological analysis. ELISA, biochemical testing, and RT-PCR analysis were performed on the six remaining brain tissues (Hamdan et al., 2022). The first portion of each fresh brain was homogenized in ice-cold Tris-HCl 50 mM (pH 7.4) (10% W/V) supplemented with 300 mM sucrose to attain 10% homogenate (w/v), which was then centrifuged at 1800 *g* for 10 min at 4 °C to obtain the supernatant for biochemical measurements. The second portion of the brain tissues was rapidly frozen at −80 °C until it was used for RT-PCR analyses (Chou et al., 2021; Mohammadi et al., 2021).

### 2.7 Biochemical estimations

#### 2.7.1 Colorimetric assays

The brain tissue homogenates were examined for oxidative stress indicators in the brain tissues, as previously reported (Abu-Elfotuh et al., 2025). Commercially available colorimetric assay kits were used to measure malondialdehyde (MDA), superoxide dismutase (SOD), and total antioxidant capacity (TAC) (Novus Biologicals and Biotechne Brand®, Cairo, Egypt) with catalogue numbers NBP3-25814, NBP3-24519, and NBP3-24484, respectively. Furthermore, colorimetric analysis tested brain tissue homogenates at wavelengths of 532, 540, and 660 nm for MDA, SOD, and TAC, respectively (Morris, 1984; Cleal et al., 2021).

#### 2.7.2 ELISA

ELISA kits (Ray Biotech, Inc. Cat No: IQR-IL1b and My BioSource, Inc., San Diego, CA, United States Cat No: MBS175904) were used to estimate the inflammatory markers GSK3β (Catalog # MBS766198), IL-1β (Catalog # MBS2023030), and TNF-α (Catalog # MBS2507393) in the brain tissue homogenate. Furthermore, BDNF (Catalog # MBS824814), NF-κβ (Catalog # MBS287521), Wnt3a (Catalog # MBS2025504), CHI3L1 (Catalog # MBS7726241), and β-catenin (Catalog # MBS261324) were among the biomarkers of cognition and the degree of neurodegeneration that were estimated using ELISA kits (MyBioSource, Inc., San Diego, CA, United States).

#### 2.7.3 Fluorometric assay

Brain monoamines, such as dopamine (DA), NE, and serotonin (5-HT), were assessed using a fluorometric assay, according to a previously described method (Ali et al., 2021). Monoamines were first oxidized to their “adrenochromes” and then rearranged to their “adrenolutins,” which were detected fluorometrically. For DA, NE, and 5-HT, samples were taken at λex/λem 320/385, 385/485, and 360/470 nm, respectively. The concentrations in nanograms per gram of fresh tissue were calculated using the fluorescence of standard solutions (Hamdan et al., 2022).

### 2.8 Analysis of histopathology

Under deep anesthesia, the rats were rinsed with an isotonic NaCl solution, as previously mentioned (Hamdan et al., 2022). Four brain tissue samples from each group (n = 4/group) were fixed in 10% formalin. They were then dispersed across the hearth using 1% formaldehyde. Then, we split their heads, removed their brains, and immediately placed them on ice. After that, we made coronal and sagittal cuts to their brains and each slice in 1% formaldehyde for 2 days. Standard hematoxylin–eosin was used to stain the fixed slices, which were then allowed to air dry before being examined using photomicrographs and a light microscope with total magnifications of 400× and 100× (Liu et al., 2019; Hamdan et al., 2022; Mountaki et al., 2021).

### 2.9 RT-qPCR

Using Applied Biosystems StepOnePlus equipment, RT-qPCR was used to measure the transcripts of *AKT*, *AMPK*, *Bax*, *Beclin-1*, *Bcl-2*, *caspase-1*, *CHOP*, *CREB*, *GRP78*, *HO-1*, *m-TOR*, *NF-κB*, *NLRP3*, *Nrf2*, *PERK*, *PI3K*, *SIRT-1*, *TLR4*, and *TrKB* in rat brain tissue. Following the manufacturer’s instructions, total RNA was extracted using a Qiagen tissue extraction kit (Qiagen, Germantown, MD, United States). A sense rapid cDNA synthesizer kit (Cat No. BIO-65053) was used to reverse-transcribe the retrieved mRNA. Applied Biosystems software version 3.1 (StepOne^TM^, Waltham, MA, United States) was used to analyze the data. Table 1 displays the primer set sequencing. The formula 2^−ΔΔCT^ was used to determine the relative expression of the target genes (Liva et al., 2001).

**TABLE 1:** List of primer sequence sets utilized in rat tissues for RT-qPCR analysis.

Gene	​	Forward primer	Reverse primer
*AKT*	NM_033230	F: 5′-CGC​CTG​CCC​TTC​TAC​AAC​C-3′	R: 5′-TCA​TAC​ACA​TCT​TGC​CAC​ACG​A-3′
*AMPK*	NM_023991.2	F: 5′− AAA​GAA​CCC​TAG​CCT​GAA​GAG​G-3′	R: 5′-ACC​TTC​CGA​GAT​GAA​TGC​TTT​T-3′
*BAX*	NM_017059.2	F: 5′-GTT​GCC​CTC​TTC​TAC​TTT​G-3′	R: 5′-AGC​CAC​CCT​GGT​CTT​G-3′
*Beclin-1*	NM_001034117.1	F: 5′-AGC​ACG​CCA​TGT​ATA​GCA​AAG​A-3′	R: 5′-GGA​AGA​GGG​AAA​GGA​CAG​CAT-3′
*Bcl-2*	NM_016993.2	F: 5′-CGG​GAG​AAC​AGG​GTA​TGA-3′	R: 5′-CAG​GCT​GGA​AGG​AGA​AGA​T-3′
*CASP-1*	NM_012762.3	F: 5′-GAA​CAA​AGA​AGG​TGG​CGC​AT-3′	R: 5′-GAG​GTC​AAC​ATC​AGC​TCC​GA-3′
*CHOP*	NM_001109986.1	F: 5′-TCT​GCC​TTT​CGC​CTT​TGA​G-3′	R: 5′-GCT​TTG​GGA​GGT​GCT​TGT​G-3′
*CREB*	NM_012762	F: 5′-CAG​ACA​ACC​AGC​AGA​GTG​GA-3′	R: 5′-CTG​GAC​TGT​CTG​CCC​ATT​G-3′
*GRP78*	NM_013083.2	F: 5′-GAC​ATC​AAG​TTC​TTG​CCG​TT-3′	R: 5′-CTC​ATA​ACA​TTT​AGG​CCA​GC-3′
*HO-*1	NM_012580.2	F: 5′-CAC​CAG​CCA​CAC​AGC​ACT​AC-3′	R: 5′-CAC​CCA​CCC​CTC​AAA​AGA​CA-3′
m*-TOR*	NM_019906.2	F: 5′-GGG​CGT​AGC​GAT​AAT​GGA​G-3′	R: 5′-TGC​CGT​CAT​CTG​TCT​TTC​C-3′
*NF-κB*	NM_001276711	F: 5′-TTC​CTC​AGC​CAT​GGT​ACC​TC-3′	R: 5′-CCC​CAA​GTC​TTC​ATC​AGC​AT-3′
*NLRP3*	NM_001191642.1	F: 5′-TGC​ATG​CCG​TAT​CTG​GTT​GT-3′	R: 5′-ACC​TCT​TGC​GAG​GGT​CTT​TG-3′
*Nrf2*	NM_001399173	F: 5′-CTC​TCT​GGA​GAC​GGC​CAT​GAC​T-3′	R: 5′-CTG​GGC​TGG​GGA​CAG​TGG​TAG​T-3′
*PERK*	NM_031599.2	F: 5′-GCC​GAT​GGG​ATA​GTG​ATG-3′	R: 5′-GCA​GCC​TCT​ACA​ATG​TCT​TCT-3′
*PI3K*	NM_031789	F: 5′-GCC​CAG​GCT​TAC​TAC​AGA​C-3′	R: 5′-AAG​TAG​GGA​GGC​ATC​TCG-3′
*SIRT-1*	NM_001414959	F: 5′- GGC​ACC​GAT​CCT​CGA​ACA​AT-3′	R: 5′-CGC​TTT​GGT​GGT​TCT​GAA​AGG-3′
TLR4	NM_019178.2	F: 5′-TCA​GCT​TTG​GTC​AGT​TGG​CT-3′	R: 5′-GTC​CTT​GAC​CCA​CTG​CAA​GA-3′
TrkB	NM_012731	F: 5′- CCT​CCA​CGG​ATG​TTG​CTG​A-3′	R: 5′-GGC​TGT​TGG​TGA​TAC​CGA​AGT​A-3′
Β-Actin	NM_031144.3	F: 5′-CCG​TAA​AGA​CCT​CTA​TGC​CA-3′	R: 5′-AAG​AAA​GGG​TGT​AAA​ACG​CA-3′

### 2.10 Statistical analysis

The data were presented as mean ± SEM. The significance level was set at *p* < 0.05. Multiple comparisons were performed using one-way ANOVA and the Tukey–Kramer *post hoc* test. Statistical analysis and graph creation were conducted using GraphPad Prism, version 8.0.2 (GraphPad Software, San Diego, CA, United States). The independence of observations was confirmed to have no clustering or repeated measures within the groups. Furthermore, the study design had neither cross-contamination nor dependent variables. The assumption of homogeneity of variances (Levene’s test) and normality (Shapiro–Wilk test) were confirmed before one-way ANOVA. The nonparametric Kruskal–Wallis test and Dunn’s *post hoc* test were used to assess data that defied these presumptions. The experimental design (different cages for each animal during the testing period) guaranteed the independence of observations.

## 3 Results

### 3.1 Protective effects of Se-NPs, Ph&M alone, or their combination on the behavioral changes detected in rats with SI-induced depression

**FIGURE 1:**
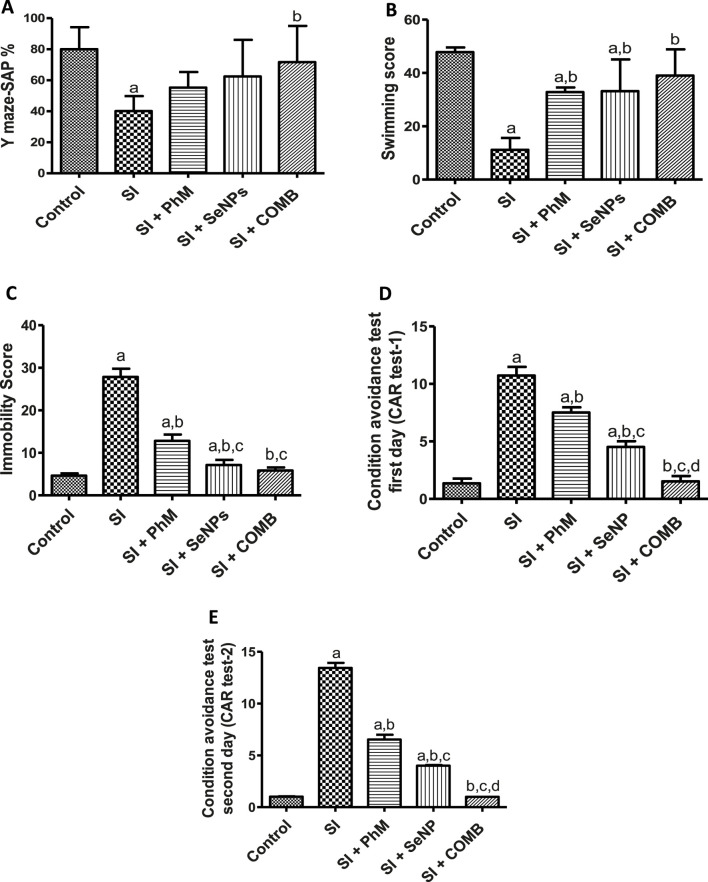
Protection effects of Ph&M, Se-NPs alone, or in combination on behavioral changes and brain proinflammatory and inflammatory biomarker levels. **(A)** Y-maze (SAP) test, **(B)** swimming score, **(C)** immobility score, **(D)** CAR test day 1, and **(E)** CAR test day 2. Data are presented as means ± SD (group n = 6). (a) Statistically significant difference relative to the normal control group. (b) Statistically significant difference relative to the SI group. (c) Statistically significant difference relative to the Ph&M group. (d) Statistically significant difference relative to the Se-NP group. Regarding parametric data, one-way ANOVA was implemented and then Tukey’s multiple comparisons test was used to evaluate the variation between groups; the significance level was set at p < 0.05. For nonparametric data, the Kruskal–Wallis test was performed, followed by Dunn’s *post hoc* multiple comparison tests.

In the Y-maze test, compared to normal control rats, SI significantly diminished SAP% by 50% (Figure 1A). However, Ph&M, Se-NPs, and COMB gradually increased SAP% by 37.5%, 55%, and 80%, respectively, without a significant difference relative to the SI group; only the COMB group showed a significant difference relative to the SI group. Upon comparing the three protected groups, no significant difference was detected.

Upon investigating the swimming score (Figure 1B), the SI group had a significantly lower swimming score of 76.5% compared with the control group. The Ph&M, Se-NPs, and COMB groups had significantly elevated swimming scores of 192.8%, 196.4%, and 248.2, respectively, compared with the SI group. No significant difference was detected among the three protected groups. All groups showed significant differences compared with the control group, except the COMB group, which had a normal score and was comparable with the control group.

Compared with the control group results, rats protected by Ph&M, Se-NPs, and COMB showed a significant decrease in the immobility score by 54, 74.1, and 79.1, respectively (Figure 1C), implying behavioral depression in socially isolated rats. The combination group showed the highest efficacy, where the normal level was retained without a significant difference compared with the control group. Upon comparing the three treated groups, the Se-NP and COMB groups had significantly decreased immobility scores compared with the Ph&M group.

The CAR test was performed in this study. The results revealed that, compared with the control group, the SI group elevated the avoidance response number to the electric shock on the first day by 7.7-fold (Figure 1D). On the second day, the number of responses increased 13.5-fold (Figure 1E), and the difference between the two groups was statistically significant on both the first and second days. Protection by Ph&M, Se-NPs, and COMB reduced the number of trials for electric shock avoidance response by approximately 30.1%, 58.1%, and 86%, respectively, on the first day and by approximately 52%, 70%, and 92.5%, respectively, on the second day compared with the SI group. There were significant differences regarding the reduced number of trials of electric shock avoidance response on the first and second days among the three protected groups. The combination group showed the highest efficacy, where the normal level was retained without a significant difference compared with the normal group.

### 3.2 Protective effects of Se-NPs, Ph&M alone, or in combination on the levels of brain neurotransmitter in rats with SI-induced depression

**FIGURE 2:**
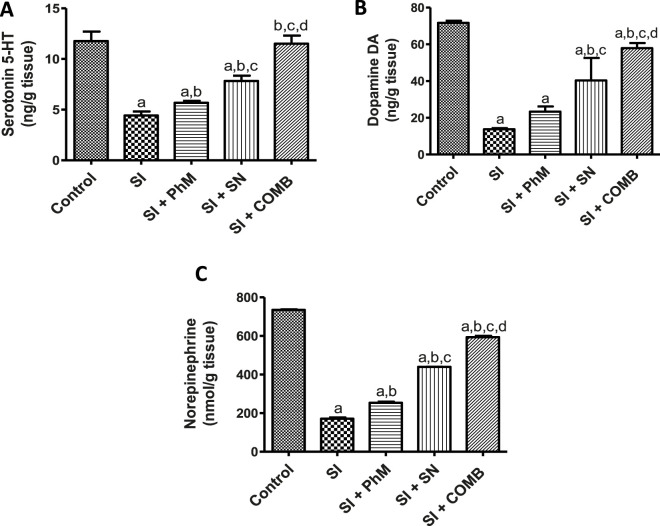
Effects of Ph&M, Se-NPs, and COMB on brain neurotransmitter levels in SI-depressed rats. **(A)** 5-HT, **(B)** DA, and **(C)** NE. The data are presented as means ± SD (group n = 6). (a) Statistically significant difference relative to the normal control group. (b) Statistically significant difference relative to the SI group. (c) Statistically significant difference relative to the Ph&M group. (d): Statistically significant difference relative to the Se-NP group. Regarding parametric data, one-way ANOVA was implemented, and then Tukey’s multiple comparisons test was used to evaluate the variation between groups; the significance level was set at p < 0.05. For nonparametric data, the Kruskal–Wallis test was performed, followed by Dunn’s *post hoc* multiple comparison tests.

Socially isolated depressed rats displayed a significant decline (p < 0.05) in the brain neurotransmitters serotonin (Figure 2A), DA (Figure 2B), and NE (Figure 2C) by 37.3%, 19.4% and 23.3%, respectively, compared with the control group. However, protecting rats with Ph&M, Se-NPs and COMB significantly increased (p < 0.05) the brain monoamines by about 48.3%, 66.1%, and 97.5%, respectively, for serotonin and 34.5%, 60.8%, and 80.8%, respectively, for NE compared with the SI group. Regarding DA, significant elevation occurred with Se-NPs and COMB (56.6% and 80.8%, respectively), and the least non-significant DA elevation (32.9%) occurred with Ph&M. COMB group showed the greatest improvement in the levels of all the tested monoamines. Upon comparing the three protected groups, significant differences were detected among all of them.

### 3.3 Protective effects of Se-NPs, Ph&M alone, or in combination on brain oxidative stress, lipid peroxidation, and cytoprotective biomarker levels in rats with SI-induced depression

**FIGURE 3:**
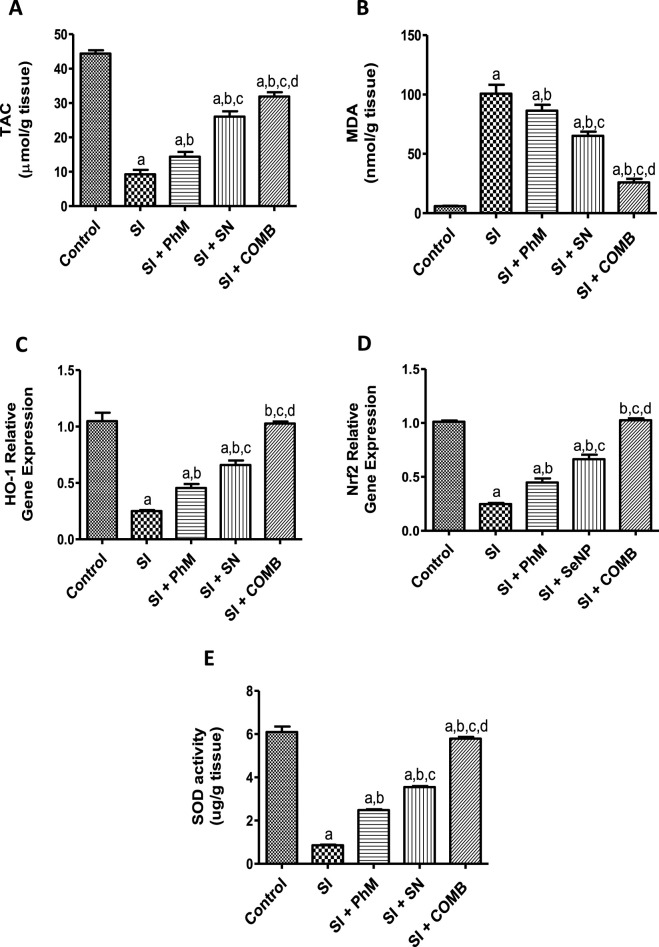
Effects of Ph&M, Se-NPs, and COMB on brain oxidative stress, lipid peroxidation, and cytoprotective biomarker levels. **(A)** TAC, **(B)** MDA, **(C)** *HO-1*, **(D)** *Nrf2*, and **(E)** SOD. Data are presented as means ± SD (group n = 6). (a) Statistically significant difference relative to the normal control group. (b) Statistically significant difference relative to the SI group. (c) Statistically significant difference relative to Ph&M group. (d) Statistically significant difference relative to the Se-NP group. Regarding parametric data, one-way ANOVA was implemented, and then Tukey’s multiple comparisons test was used to evaluate the variation between groups; the significance level was set at p < 0.05. For nonparametric data, the Kruskal–Wallis test was performed, followed by Dunn’s *post hoc* multiple comparison tests.

SI dramatically abridged the brain’s oxidative stress biomarker, TAC (Figure 3A), by about 78.7% and elevated the brain lipid peroxidation biomarker level (MDA) (Figure 3B) by about 17-fold compared with those in the control group.

Compared with the SI rats, the rats protected with Ph&M, Se-NPs, and COMB exhibited significantly higher TAC (14.4%, 58.8%, and 71.8%, respectively) and significantly lower MDA levels by about 1.2-, 1.5-, and 3.9-fold, respectively. It was found that COMB group had a remarkable protective influence on oxidative stress indicators, mostly TAC, associated with perceptible reducing influences on MDA.

Moreover, the SI group displayed substantial statistically significant reductions in *HO-1* (Figure 3C) and *Nrf2* (Figure 3D) concentrations and SOD activity (Figure 3E) by 76.2%, 75.2%, and 85.2%, respectively, compared with the results of the normal control group. Protection with Ph&M, Se-NPs, or their combination significantly restored the amounts of HO-1, Nrf2, and SOD to approximately levels compared with the results of the SI group. Upon comparing protection by Ph&M and Se-NPs, the COMB group had the highest impact on HO-1 and Nrf2 levels and SOD activity with a significant difference.

It was observed that the protective outcome of Se-NPs was significantly greater than that of Ph&M, indicating that Se-NPs have a protective effect against SI-induced oxidative stress by lowering the peroxidation of lipids and the levels of the *Nrf2* biomarker. The combination of Ph&M and Se-NPs synergistically protected against the declines in lipid peroxidation and oxidative stress biomarkers. These results showed that either single or combination protection could inhibit oxidative stress, lipid peroxidation, and cytoprotective biomarker levels, especially in combination, which restored the level of the markers to almost normal values.

### 3.4 Protective effects of Se-NPs, Ph&M alone, or in combination on brain proinflammatory and inflammatory biomarker levels in rats with SI-induced depression

**FIGURE 4:**
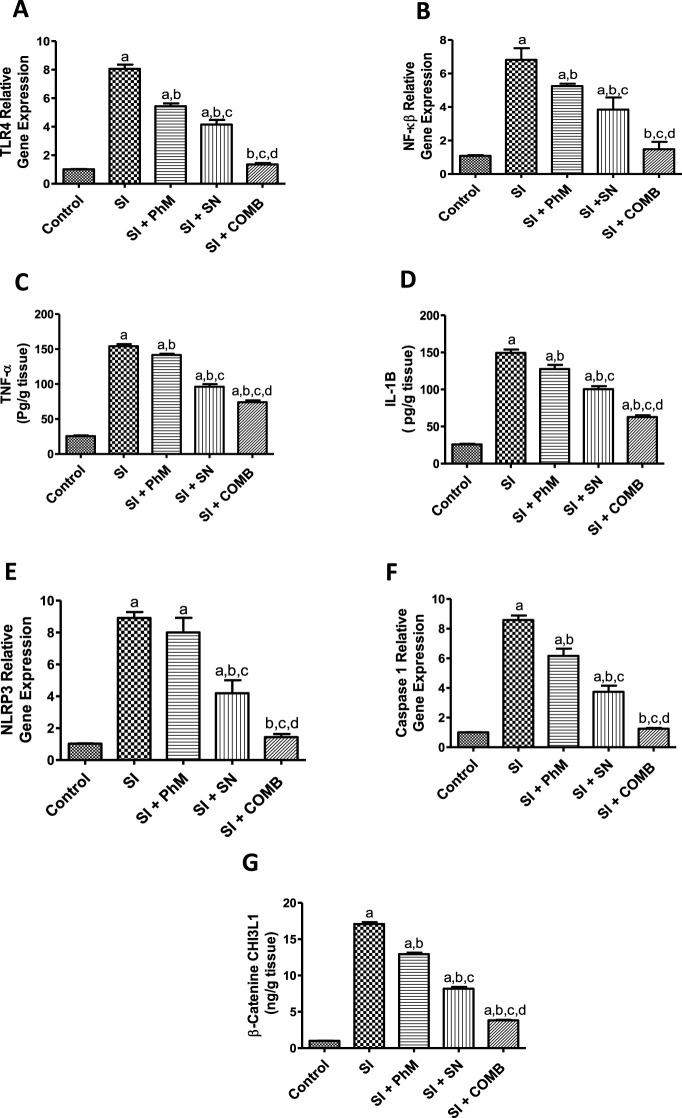
Protective effects of Ph&M, Se-NPs alone, or in combination on brain proinflammatory and inflammatory biomarker levels. **(A)** TLR4, **(B)** NF-κB, **(C)** TNF-α, **(D)** IL-1β, **(E)** NLRP3, **(F)** caspase-1, and **(G)** CHI3L1. Data are presented as means ± SD (group n = 6). (a) Statistically significant difference relative to the normal control group. (b) Statistically significant difference relative to the SI group. (c) Statistically significant difference relative to the Ph&M group. (d) Statistically significant difference relative to the Se-NP group. Regarding parametric data, one-way ANOVA was implemented, and then Tukey’s multiple comparisons test was used to evaluate the variation between groups; the significance level was set at p < 0.05. For nonparametric data, the Kruskal–Wallis test was performed, followed by Dunn’s *post hoc* multiple comparison tests.

The expression of the genes that regulate inflammatory responses, *TLR4* (Figure 4A) and *NF-κB* (Figure 4B), was markedly elevated in SI rats compared with that of the controls (p < 0.05). Ph&M, Se-NPs, and COMB significantly reduced the levels of the tested parameters compared with the results of the SI group by about 32.6%, 48.5%, and 83.3%, respectively, for TLR4 and 23%, 43.5%, and 78%, respectively, for NF-κB.

Therefore, compared with Ph&M, Se-NPs showed a stronger protective impact against SI-induced elevations in TLR4 and NF-κB levels. The rise in TLR4 and NF-κB levels was also influenced synergistically by the Ph&M and Se-NP combination. Furthermore, compared with the Ph&M and Se-NPs groups, combination therapy significantly affected all evaluated neuroinflammatory biomarkers.

Compared with the results of the control group, SI significantly increased the brain neuroinflammatory levels of *TNF-α* (Figure 4C) and *IL-1β* (Figure 4D) by approximately 500% and 477%, respectively. In contrast, pretreatment with Ph&M, Se-NPs, or their combination significantly decreased brain IL-1β levels by 72%, 67.2%, and 42%, respectively, and TNF-α levels by approximately 91.7%, 62.4%, and 48.1%, respectively. These results demonstrated the valuable protective effect of Ph&M, Se-NPs, and COMB against SI-induced elevations in inflammatory biomarker levels, and the combination of Ph&M and Se-NPs had a synergistic effect on all measured biomarker levels.

Compared with normal control rats, SI rats displayed nearly maximal elevations in the values of the inflammasome *NLRP3* (Figure 4E) and *caspase-1* (Figure 4F) by 790% and 760%, respectively. NLRP3 levels were significantly reduced by about 47.23% and 15.7% in the Se-NP and combination protected groups, respectively, while a slight reduction of 89.9% occurred in the Ph&M-protected groups.

Ph&M, Se-NPs, and COMB significantly reduced the values of caspase-1 by 27.9%, 57%, and 84.3%, respectively, in the SI group. The protective effect of Se-NPs against caspase-1 was significantly higher than that of Ph&M. However, upon combining Ph&M and Se-NPs, caspase-1 levels returned to normal without a significant difference compared with the normal control group. Combination therapy had the highest recovery influence on the NLRP3 and caspase-1 levels.

Regarding the inflammatory biomarker CHI3L1, the SI group displayed a 17.5-fold elevation in CHI3L1 protein expression (Figure 4G). Ph&M, Se-NPs, and their combination significantly abridged this increase by 24.2%, 52.2%, and 77.6%, respectively. It was observed that the maximum decline in CHI3L1 was achieved by the combination treatment.

### 3.5 Protective effects of Se-NPs, Ph&M alone, or in combination on ER stress, autophagy, apoptosis, and neurodegenerative biomarker levels in rats with SI-induced depression

**FIGURE 5:**
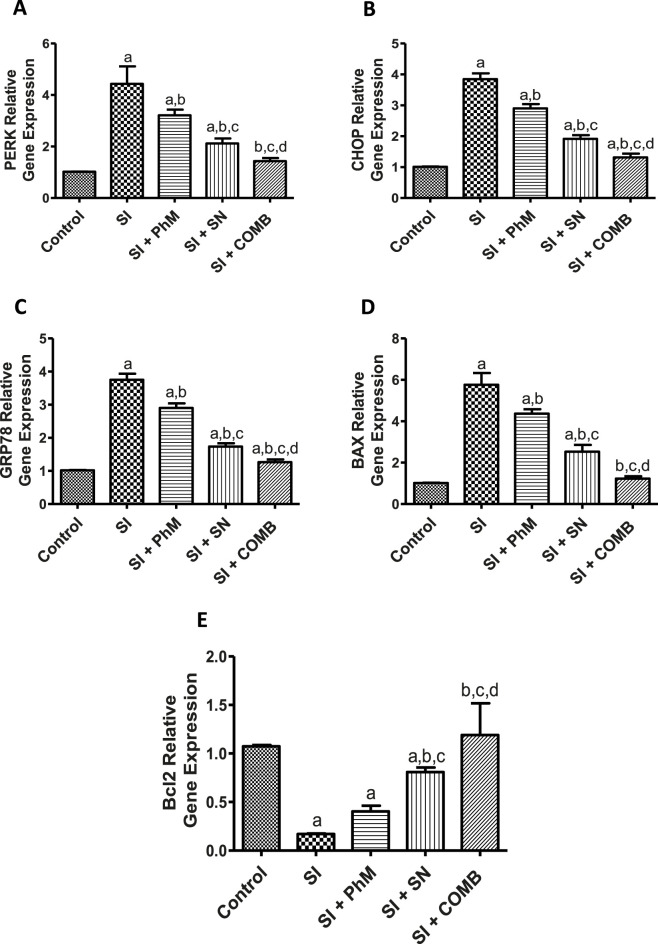
Protection effects of Ph&M, Se-NPs alone, or in combination on brain ER stress, autophagy, apoptosis, and neurodegenerative biomarkers. **(A)** PERK, **(B)** CHOP, **(C)** GRP78, **(D)** Bax, and **(E)** Bcl-2. Data are presented as means ± SD (group n = 6). (a) Statistically significant difference relative to the normal control group. (b) Statistically significant difference relative to the SI group. (c) Statistically significant difference relative to the Ph&M group. (d) Statistically significant difference relative to the Se-NP group. Regarding parametric data, one-way ANOVA was implemented, and then Tukey’s multiple comparisons test was used to evaluate the variation between groups; the significance level was set at p < 0.05. For nonparametric data, the Kruskal–Wallis test was performed, followed by Dunn’s *post hoc* multiple comparison tests.

The estimated autophagy and ER stress parameters are displayed in Figure 4. Rat depression brought on by SI significantly changed the levels of ER stress markers, according to assessments of PERK (Figure 5A), CHOP (Figure 5B), and GRP78 (Figure 5C). Furthermore, combination therapy has the greatest effect on boosting autophagy and reducing ER stress, both of which function in concert to protect against the negative effects of SI-induced depression. As a result, Ph&M and Se-NPs worked in concert to protect against higher levels of all assessed neurodegenerative biomarkers and cognitive deficits.

Furthermore, the abnormal pro-apoptotic effect on brain cells was exceptionally high in SI animals. This result was confirmed by the downregulation of the anti-apoptotic Bcl-2 (Figure 5E) (84% decrease) and the overexpression of the apoptotic regulator Bax (Figure 5D) (5.8-fold increase). Notably, by rectifying the imbalance in apoptotic markers, protection by Ph&M, Se-NPs alone, or in combination significantly reduced apoptosis. The combined therapy was the most effective at inhibiting apoptosis.

It is interesting to note that Ph&M and Se-NPs together provide a strong defense against elevated levels of every assessed biomarker of neurodegeneration and cognitive impairment.

### 3.6 Protective effects of Se-NPs, Ph&M alone, or in combination on the AMPK/SIRT-1/Beclin-1 pathway in rats with SI-induced depression

**FIGURE 6:**
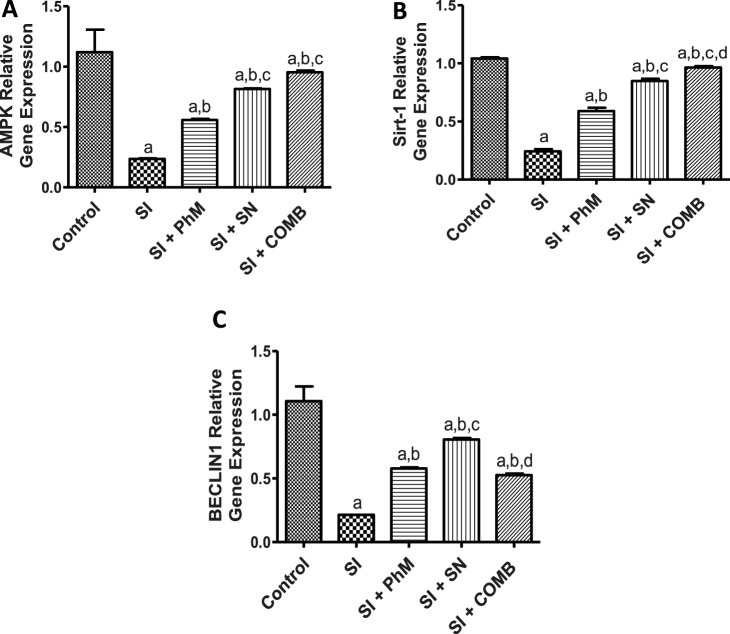
Protection effects of Ph&M, Se-NPs alone, or in combination on the AMPK/SIRT-1/Beclin-1 pathway in rats with SI-induced depression. **(A)** AMPK, **(B)** SIRT-1, and **(C)** Beclin-1. Data are presented as means ± SD (group n = 6). (a) Statistically significant difference relative to the normal control group. (b) Statistically significant difference relative to the SI group. (c) Statistically significant difference relative to the Ph&M group. (d) Statistically significant difference relative to the Se-NP group. Regarding parametric data, one-way ANOVA was implemented, and then Tukey’s multiple comparisons test was used to evaluate the variation between groups; the significance level was set at p < 0.05. For nonparametric data, the Kruskal–Wallis test was performed, followed by Dunn’s *post hoc* multiple comparison tests.

Compared with the healthy control group, SI significantly decreased the mRNA levels of AMPK, SIRT-1, and Beclin-1 by 78.6%, 80.9%, and 76%, respectively (Figure 6). The mRNA levels of the animals protected with Ph&M, Se-NPs, and COMB were significantly upregulated compared with those of SI animals. The combination of Ph&M and Se-NPs showed the highest protective effect, except for Beclin-1, where the combination exhibited the least efficacy.

### 3.7 Protective effects of Se-NPs, Ph&M alone, or in combination on the PI3K/AKT/m-TOR pathway in rats with SI-induced depression

**FIGURE 7:**
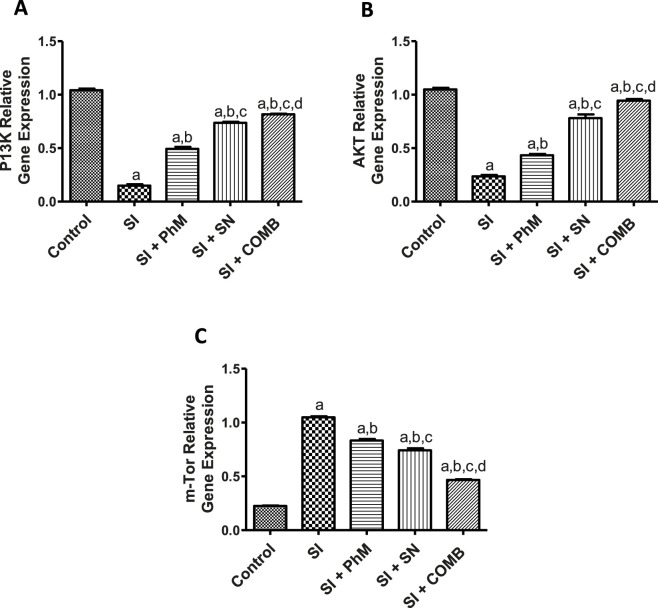
Protection effects of Ph&M, Se-NPs alone, or in combination on the PI3K/AKT/m-TOR pathway in rats with SI-induced depression. **(A)** PI3K, **(B)** AKT, and **(C)** m-TOR. Data are presented as means ± SD (group n = 6). (a) Statistically significant difference relative to the normal control group. (b) Statistically significant difference relative to the SI group. (c) Statistically significant difference relative to the Ph&M group. (d) Statistically significant difference relative to the Se-NP group. Regarding parametric data, one-way ANOVA was implemented, and then Tukey’s multiple comparisons test was used to evaluate the variation between groups; the significance level was set at p < 0.05. For nonparametric data, the Kruskal–Wallis test was performed, followed by Dunn’s *post hoc* multiple comparison tests.

The results showed that *PI3K* and *AKT* mRNA levels (Figure 7) were significantly downregulated by 85.6% and 77.1%, respectively, in the SI group compared with those of the control group, while m-TOR mRNA levels were significantly upregulated by 4.6-fold in the SI group. However, these alterations were substantially reversed following the use of Ph&M, Se-NPs, and COMB, where Ph&M significantly downregulated *m-TOR* mRNA levels by 20.9% and significantly upregulated PI3K and AKT mRNA levels by 4.9- and 1.9-fold, respectively, in comparison with those of the SI group.

Similarly, compared to the SI group, Se-NP therapy significantly downregulated m-TOR mRNA levels by 29.5% and significantly increased PI3K and AKT mRNA levels (4.5- and 3.3-fold respectively). It was observed that the highest efficacy was detected among the Ph&M and Se-NP combination group where PI3K and AKT mRNA levels were significantly upregulated by 5.5- and 3.9-fold, respectively, and m-TOR mRNA levels were significantly downregulated by 55.2% compared with the SI group.

### 3.8 Protective effects of Se-NPs, Ph&M alone, or in combination on the CREB/BDNF/TrkB pathway in rats with SI-induced depression

**FIGURE 8:**
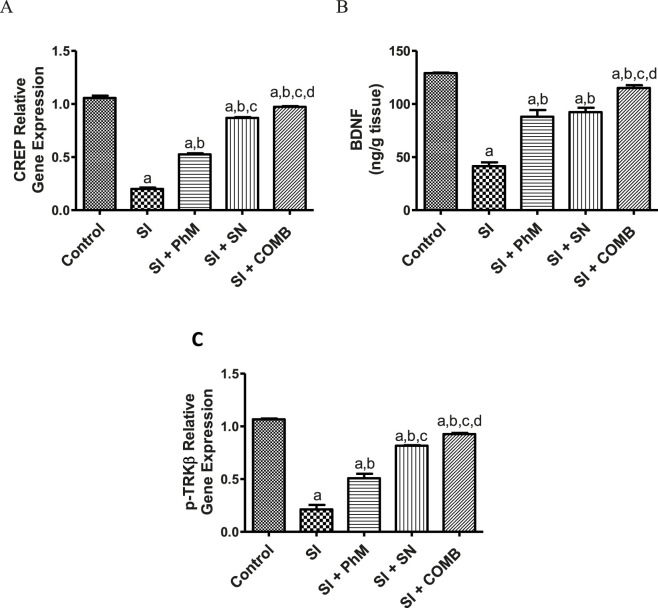
Protection effects of Ph&M, Se-NPs alone, or in combination on the CREB/BDNF/TrkB pathway. **(A)** CREB, **(B)** BDNF, and **(C)** TrkB. Data are presented as means ± SD (group n = 6). (a) Statistically significant difference relative to the normal control group. (b) Statistically significant difference relative to the SI group. (c) Statistically significant difference relative to the Ph&M group. (d) Statistically significant difference relative to the Se-NP group. Regarding parametric data, one-way ANOVA was implemented, and then Tukey’s multiple comparisons test was used to evaluate the variation between groups; the significance level was set at p < 0.05. For nonparametric data, the Kruskal–Wallis test was performed, followed by Dunn’s *post hoc* multiple comparison tests.

Furthermore, compared with control rats, SI significantly downregulated TrkB and CREB by 4.7- and 5.3-fold, respectively. In contrast to the rat group with SI-induced depression, protection by Ph&M, Se-NPs, and their combination dramatically increased the expression of TrkB and CREB mRNA (Figures 8A,C) (by 2.6-, 4.1-, and 4.9-fold, respectively, for TrkB and by 2.5-, 4.4-, and 4.8-fold, respectively, for CREB). Rats in the SI group showed a significant 68% drop in BDNF activity in their brains compared to those in the control group (Figure 3B).

In contrast, protection with Ph&M, Se-NPs, and their combination dramatically reversed the decreases in BDNF levels by roughly 112.4%, 122%, and 177.2%, respectively, compared to the SI group. The combined effect on BDNF activity in the brain was comparable between the Se-NPs- and Ph&M-protected groups.

### 3.9 Protective effects of Se-NPs, Ph&M alone, or in combination on the Wnt3/β-catenin/GSK3β signaling pathway in rats with SI-induced depression

**FIGURE 9:**
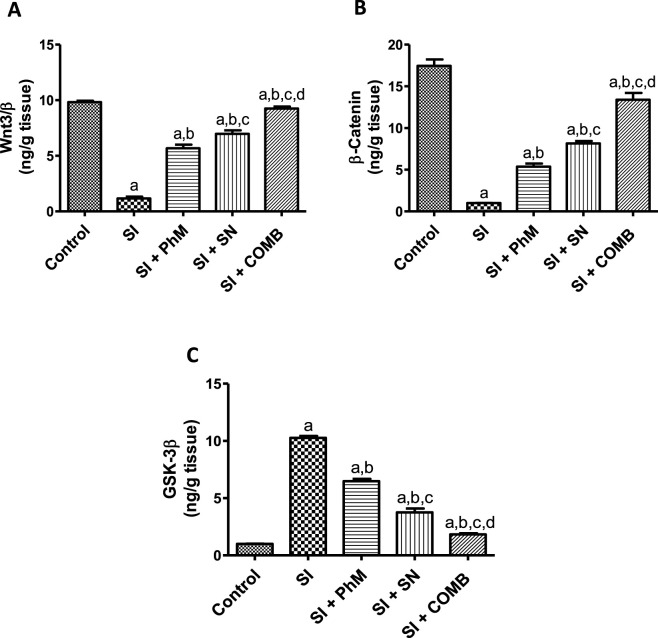
Effects of Ph&M, Se-NPs, and COMB on the Wnt3/β-catenin/GSK3β signaling pathway. **(A)** Wnt3a, **(B)** β-catenin, and **(C)** GSK3β levels. Data are presented as means ± SD (group n = 6). (a) Statistically significant difference relative to the normal control group. (b) Statistically significant difference relative to the SI group. (c) Statistically significant difference relative to the Ph&M group. (d) Statistically significant difference relative to the Se-NP group. Regarding parametric data, one-way ANOVA was implemented, and then Tukey’s multiple comparisons test was used to evaluate the variation between groups; the significance level was set at p < 0.05. For nonparametric data, the Kruskal–Wallis test was performed, followed by Dunn’s *post hoc* multiple comparison tests.

Compared with the results of the control group, Figures 9A–C demonstrates a significant drop in Wnt3a and β-catenin levels of 87.8% and 94.2%, respectively, along with a 10.2-fold increase in GSK3β activity. However, these results were significantly reversed with Ph&M, Se-NPs, and COMB compared with those of the SI group. For Wnt3a, the elevation was 4.8-, 5.8-, and 7.8-fold, respectively, for β-catenin and 5.3-, 8.2-, and 13.1-fold, respectively, for GSK3β, and the reduction was 36.9%, 63.4%, and 82.2%, respectively.

Compared with the effects of Ph&M and Se-NPs alone, COMB protection exhibited the greatest ameliorating activity for the restoration of the Wnt3/β-catenin/GSK3β signaling pathway. Using Ph&M, Se-NPs, and COMB significantly inhibited the SI downregulation of Wnt3a and β-catenin. In the meantime, all three groups showed significant inhibition of GSK3β activity.

### 3.10 Effects of Ph&M, Se-NPs, and COMB on histopathological changes in the brain of socially isolated depressed rats

**FIGURE 10:**
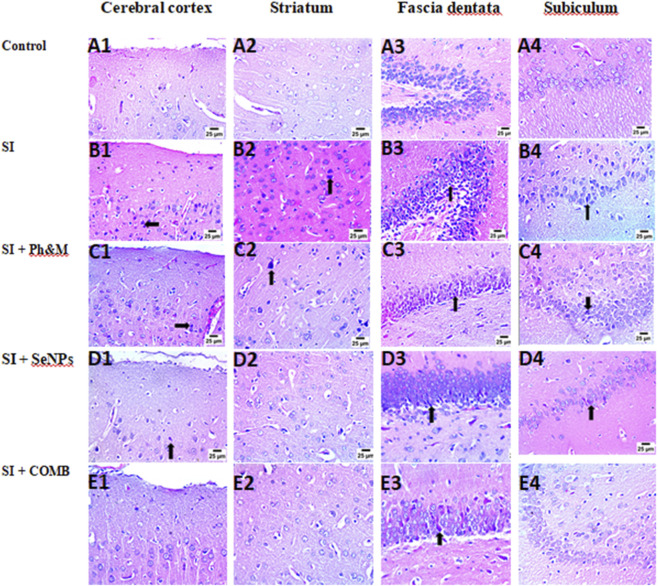
Effects of Ph&M, Se-NPs, and COMB on histopathological changes in the brains of SI depressed rats. **(A)** Control, **(B)** SI, **(C)** SI + Ph&M, **(D)** SI + Se-NPs, and **(E)** SI + COMB. Nuclear pyknosis in brain sections is indicated by black arrows.

Figure 10 shows a Photomicrograph of the normal histologic structures of the cerebral cortex, striatum, fascia dentate, and subiculum in the normal control group (Figures 10A1–A4). However, the SI group showed a moderate number of degenerated and shrunken neurons with severe nuclear pyknosis (black arrow) in the cerebral cortex (Figure 10B1), mild nuclear pyknosis (black arrow) was detected in the striatum (Figure 10B2), and severe nuclear pyknosis (black arrow) was found in the neurons of the fascia dentata (Figure 10B3). In addition, mild nuclear pyknosis (black arrow) appeared in the subiculum (Figure 10B4).

In contrast, Ph&M showed a few degenerated neurons with mild nuclear pyknosis (black arrow) in the cerebral cortex (Figure 10C1) and mild nuclear pyknosis in the striatum (Figure 10C2). Severe nuclear pyknosis (black arrow) was also observed in the neurons of the fascia dentata (Figure 10C3), while mild nuclear pyknosis (black arrow) was detected in the subiculum (Figure 109C4).

In contrast, Se-NPs showed a small number of degenerated neurons with mild nuclear pyknosis (black arrow) in the cerebral cortex (Figure 10D1), normal histological structure of neurons (black arrow) was found in the striatum (Figure 10D2), and mild nuclear pyknosis (black arrow) was detected in the neurons of the fascia dentata and subiculum (Figures 10D3, 4).

The combination group showed the highest protection of brain tissue. with normal histological structure of neurons in the cerebral cortex, striatum, and subiculum (Figures 10E1–3); conversely, mild nuclear pyknosis (black arrow) was detected in the neurons of the fascia dentata (Figure 10E4).

**FIGURE 11:**
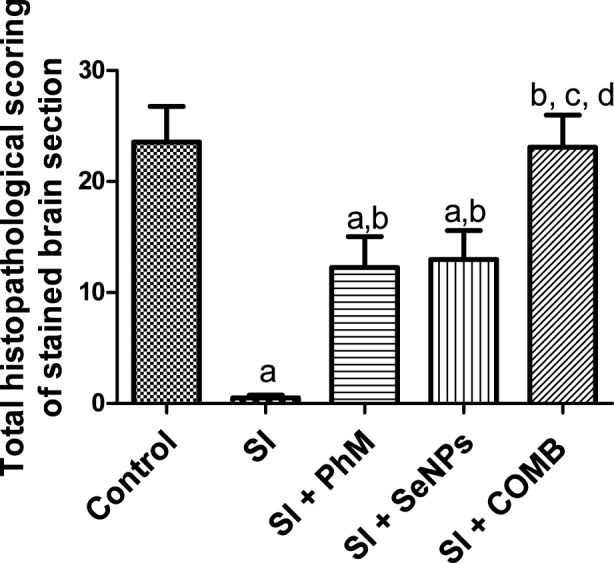
Bar chart showing the effects of Ph&M, Se-NPs, and COMB on histopathological changes in the brains of SI depressed rats on the total histopathological scoring of the stained brain section. The total histopathological scoring was recorded by the summation of histopathological scoring of each group, which was compared among different groups. Data were presented as means ± SE (n = 10); p < 0.05. Compared with the (a) control, (b) SI, (c) SI + Ph&M, and (d) SI + Se-NP groups. Statistical analysis was performed using one-way ANOVA, followed by the Tukey–Kramer *post hoc* test.

The effects of Ph&M, Se-NPs, and COMB on the total histopathological scoring of histopathological changes in the brains of SI depressed rats are illustrated in Figure 10. The brain sections of normal control rats showed normal histological structures of the cerebral cortex, striatum, fascia dentate, and subiculum. However as shown in Figure 11, the brain tissue of the SI group showed degenerated and shrunken neurons with severe nuclear pyknosis. Both the Ph&M and Se-NP groups exhibited a few degenerated neurons with mild nuclear pyknosis, with significant differences compared with the control group. Upon comparing both groups, no significant difference was detected. Interestingly, the COMB group showed a significant increase in total histopathological scoring compared with the Ph&M and Se-NP groups. It is important to mention that the COMB group restored the histopathological scoring to almost normal levels, where no significant difference was recorded compared with the normal control group.

**FIGURE 12:**
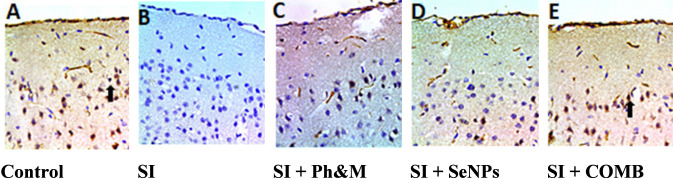
Effects of Ph&M, Se-NPs, and COMB on the expression for BDNF in the neurons of the cerebral cortex in the brains of SI depressed rats. **(A)** Control, **(B)** SI, **(C)** SI + Ph&M, **(D)** SI + Se-NPs, and **(E)** SI + COMB. Nuclear pyknosis in brain sections is indicated by black arrows.

As shown in Figure 12, severe positive expression of BDNF in the neurons of the cerebral cortex was detected in the control and combination groups, negative expression of BDNF in the neurons of the cerebral cortex was detected in the control negative, and mild negative expression of BDNF was detected in the neurons of the Ph&M and SeNPs groups.

## 4 Discussion

Depression is the most common mental disorder affecting more than 264 million people worldwide; almost 800,000 people die by suicide every year (Zolfaghari et al., 2021; Ilic and Ilic, 2022). Depression may be caused by various contributing factors, such as monoamine deficiency, neuroinflammation, neuroplastic changes, and HPA axis hyperactivity (Herbert and Lucassen, 2016).

This study aims to explore the protective effects of Se supplementation, which is one of the major trace elements involved in various antioxidant defenses (Wang J. et al., 2018) and has previously been tested against stress-induced depression (Sajjadi et al., 2022). Nanoformulation has considerable advantages in terms of pharmacological performance, safety issues, tissue biodistribution profiles, and physicochemical properties (Sari et al., 2022). The possible protective effects of Ph&M and the combination of Se-NPs and Ph&M are also assessed.

This study attempts to identify possible preventive measures to protect against depression without relying on mainstay pharmacologic therapies, mainly antidepressants, which may have devastating adverse effects (Braund et al., 2021).

Among these adverse effects is suicidal ideation, which is especially common with selective serotonin reuptake inhibitor use (Sørensen et al., 2022). Antidepressants generally manifest limited clinical efficacy and delayed onset of action (Jiang et al., 2022).

In the current study, SI exhibited a magnitude of negative effects on brain cells. SI precipitated a significant reduction in brain neurotransmitters, created oxidative stress, induced inflammation, increased ER stress, and decreased autophagy with a consequent increase in neuronal apoptosis. These changes led to neural loss, brain function disturbances, and behavioral changes comparable to depression in humans.

SI significantly increased the immobility score and elevated the avoidance response number to electric shock on the first and second days compared with those of the normal control group. SI also significantly decreased the swimming and climbing scores as well as the SAP% for the Y-maze test in comparison with those of the normal control group. These findings are consistent with previous results (Alqurashi et al., 2022) showing the effects of stress-induced depression on behavioral tests. We can conclude that in our study, SI induced behavioral symptoms similar to those of depression in humans. These symptoms can be attributed to the wholesome oxidative, inflammatory, and neural assaults shown by the histopathological and biochemical parameters measured in the current study.

Conversely, protection by Ph&M, Se-NPs, and COMB reduced the elevation immobility score and the number of trials for electric shock avoidance response caused by SI and increased the swimming and climbing scores and SAP%, with COMB showing the maximum protection. This result can be attributed to the efficacy of Ph&M, Se-NPs, and COMB in defying the underlying cellular changes caused by SI.

Depression etiology is highly attributed to the dysregulation of monoamine neurotransmitter systems (Shao and Zhu, 2020; Delgado, 2000). In the current study, SI decreased the neurotransmitters responsible for mood modulation (Liu et al., 2018). Ph&M, Se-NPs, and COMB increased the levels of all three monoamines, with COMB showing the most effectiveness, which can explain the improvements created by these proposed therapies on behavioral tests.

Other possible etiologies for depression are neuroinflammation and the presence of oxidative stress, which impairs neurogenesis and synaptic plasticity (Correia et al., 2023). In our study, SI-induced depression exhibited neuronal oxidative stress and increased free radicals, which in turn triggered lipid peroxidation and raised levels of MDA, a prominent neural oxidative stress biomarker (Abu-Elfotuh et al., 2023). Nrf2, a critical activator of antioxidant enzymes (SOD1 and HO-1) that prevents oxidative stress, suppresses inflammation mediated by microglia, and boosts mitochondrial function, was also affected by SI-induced depression. This depression subsequently altered SOD1 and HO-1, as well as TAC (Abu-Elfotuh et al., 2023). However, Ph&M, Se-NPs, and COMB remarkably boosted antioxidant capabilities, with maximum efficacy exhibited by COMB.

In the present study, our observations confirmed increased transcription of inflammatory response genes TLR4 and NF-κB, as well as the activation of inflammatory cytokines TNF-α and IL-1β in the SI-induced depression group. This confirms the activation of proinflammatory pathways, such as NLRP3/caspase-1, where NLRP3 and caspase-1 levels were maximally elevated by SI-induced depression. Ph&M, Se-NPs, and COMB markedly decreased the expression of inflammatory genes and cytokines and suppressed the NLRP3/caspase-1 pathway, again with the maximum efficacy for COMB. These findings suggest that the proposed therapies in this study can prevent neural injury, as previous studies have shown that drugs that reduce the production of pyroptosis-related proteins, including caspase-1, and inhibit the NLRP3 inflammasome may be suitable for both curative and preventive treatment of neurological diseases (Abu-Elfotuh et al., 2023; Liang et al., 2021).

ER stress parameters PERK, CHOP, and GRP78 were significantly elevated by SI. This was accompanied by the downregulation of the anti-apoptotic Bcl-2 and the overexpression of the apoptotic regulator Bax. Our proposed therapies, especially their combination, counteracted ER stress by reducing PERK, CHOP, and GRP78; upregulated Bcl-2 expression; and decreased the expression of BAX.

Our results showed that SI-induced depression leads to ER stress, as proven by elevated levels of ER stress biomarkers, including GRP78, PERK, and CHOP.

Prolonged ER stress induces neural cell death or apoptosis. The attenuation of the anti-apoptotic regulator Bcl-2 protein facilitates this process (Gao et al., 2022). Additionally, the upregulation of CHOP stimulates apoptosis.

Impairment of autophagy was exhibited by SI-induced depression, as seen in the current study via the impairment of AMPK/SIRT-1/Beclin-1, as well as PI3K/AKT/m-TOR pathways, indicating that these depression-like symptoms increase ER stress and apoptosis and impair autophagy. Impairment of autophagy prevents the removal of excessive unfolded proteins and jeopardizes cell survival, leading to neural cell loss and consequent cognitive decline (Esmaeili et al., 2022; Ekundayo et al., 2024; Wang B.-J. et al., 2018).

Once again, Se-NPs, Ph&M, and, to a greater extent, their combination provided adequate augmentation for autophagy, suggesting their capability to preserve neurons and maintain cognitive capabilities.

TrkB, as a receptor for BDNF among other neurotrophins, plays a crucial role during development, maintenance of the adult brain, and its adaptation to injury or pathological conditions (Deogracias et al., 2004). BDNF is an important neurotrophin, and its depletion is associated with neurodegenerative disorders of the central nervous system, such as Alzheimer’s disease, multiple sclerosis, and Parkinson’s disease (Bai et al., 2019).

BDNF plays a crucial role in brain development and is involved in learning and memory via the CREB/BDNF/TrkB pathway, in which BDNF binding to TrkB enhances autophosphorylation and activates CREB. CREB is necessary for multiple biological processes, such as neuron survival, differentiation, and synaptic transmission in the brain, together with further increase in BDNF gene expression (Bai et al., 2019).

In contrast to SI-induced depression in the rat group, protection by Ph&M, Se-NPs, and their combination dramatically increased the expression of TrkB and CREB mRNA with an accompanying increase in BDNF.

The Wnt/β-catenin signaling pathway has been implicated in neuronal synaptogenesis and remodeling (Pérez-Palma et al., 2016). Hampering of such pathways negatively affects neuroplasticity and cognitive functions. SI caused drastic drops in Wnt3a and β-catenin levels. However, it enormously increased GSK3β activity. The dysregulation of the Wnt3/β-catenin/GSK3β axis is linked to a wide range of illnesses, including cancer, kidney disease, bone problems, and neurodegenerative diseases (Liu et al., 2019) and the inhibition of GSK3β has benefits against neurodegeneration (Hu et al., 2009).

Se-NPs and Ph&M showed marked improvement of Wnt3/β-catenin/GSK3β signaling expressions, with their combination exhibiting the maximum efficacy, and all three proposed treatments markedly inhibited GSK3β activity.

Finally, we can conclude that Se-NPs, Ph&M, and especially their combination applied in this study could exert possible therapeutic and protective effects against neuroinflammation, oxidative stress, and apoptosis that are subsequently reflected on the behavior of test animals.

### 4.1 Limitation

We offer nominal p-values without multiple testing adjustments due to the exploratory character of this work and the numerous planned comparisons (several behavioral, biochemical, and molecular endpoints). Although this method first identifies possible signals, it may raise the possibility of a type I error. Readers should keep this in mind while interpreting the data, and results should be viewed as hypothesis-generating. Larger sample sizes and preregistered endpoints are necessary for future confirmatory research.

## 5 Conclusion

In conclusion, our study revealed that chronic exposure to SI is a precipitating factor for depression, which we have proved histologically, pharmacologically, biochemically, and behaviorally in a rat model. This can occur because of the long-term use of mobiles at a young age. We proved that the nutritional inclusion of Se and Ph&M can protect against the deleterious effects of SI (Graphical abstract).

## 6 Recommendations

The inclusion of Se-rich nutritional diets, such as nuts, seafood (such as tuna and salmon), meat (such as beef and turkey), eggs, mushrooms, and sunflower seeds, with daily Ph&M protects against social isolation induced depression that can precipitated by modern life style, including long term mobile use. Further clinical studies should be performed on different groups to establish a protocol of protection against SI-induced depression, especially in children and young people.

## Data Availability

The original contributions presented in the study are included in the article/supplementary material; further inquiries can be directed to the corresponding author.

## References

[B1] Abu-Elfotuh K. Al-Najjar A. H. Mohammed A. A. Aboutaleb A. S. Badawi G. A. (2022a). Fluoxetine ameliorates Alzheimer’s disease progression and prevents the exacerbation of cardiovascular dysfunction of socially isolated depressed rats through activation of Nrf2/HO-1 and hindering TLR4/NLRP3 inflammasome signaling pathway. Int. Immunopharmacol. 104, 108488. 10.1016/j.intimp.2021.108488 35042170

[B2] Abu-Elfotuh K. Abdel-Sattar S. A. Abbas A. N. Mahran Y. F. Alshanwani A. R. Hamdan A. M. E. (2022b). The protective effect of thymoquinone or/and thymol against monosodium glutamate-induced attention-deficit/hyperactivity disorder (ADHD)-like behavior in rats: modulation of Nrf2/HO-1, TLR4/NF-κB/NLRP3/caspase-1 and Wnt/β-Catenin signaling pathways in rat model. Biomed. and Pharmacother. 155, 113799. 10.1016/j.biopha.2022.113799 36271575

[B3] Abu-Elfotuh K. Tolba A. M. Hussein F. H. Hamdan A. M. Rabeh M. A. Alshahri S. A. (2023). Anti-alzheimer activity of combinations of cocoa with vinpocetine or other nutraceuticals in rat model: modulation of wnt3/β-catenin/GSK-3β/nrf2/HO-1 and PERK/CHOP/Bcl-2 pathways. Pharmaceutics 15 (8), 2063. 10.3390/pharmaceutics15082063 37631278 PMC10457980

[B4] Abu-Elfotuh K. Mahran Y. Bayoumie El Gazzar W. Youssef H. S. Hamdan A. M. Albalawi T. M. (2025). Targeting Ferroptosis/Nrf2 pathway ameliorates AlCl3-Induced alzheimer’s disease in rats: neuroprotective effect of morin hydrate, zeolite clinoptilolite, and physical plus mental activities. Int. J. Mol. Sci. 26 (3), 1260. 10.3390/ijms26031260 39941034 PMC11818523

[B5] Ali F. E. Sayed A. M. El-Bahrawy A. H. Omar Z. M. Hassanein E. H. (2021). Targeting KEAP1/Nrf2, AKT, and PPAR-γ signals as a potential protective mechanism of diosmin against gentamicin-induced nephrotoxicity. Life Sci. 275, 119349. 10.1016/j.lfs.2021.119349 33744325

[B6] Alqurashi G. K. Hindi E. A. Zayed M. A. Abd El-Aziz G. S. Alturkistani H. A. Ibrahim R. F. (2022). The impact of chronic unpredictable mild stress-induced depression on spatial, recognition and reference memory tasks in mice: behavioral and histological study. Behav. Sci. 12 (6), 166. 10.3390/bs12060166 35735376 PMC9219659

[B7] Arslankiran A. Acikgoz B. Demirtas H. Dalkiran B. Kiray A. Aksu I. (2025). Effects of voluntary or involuntary exercise in adolescent male rats exposed to chronic social isolation on cognition, behavior, and neurotrophic factors. Biol. Futura 76, 71–85. 10.1007/s42977-025-00250-w 39966302

[B8] Bai L. Zhang S. Zhou X. Li Y. Bai J. (2019). Brain-derived neurotrophic factor induces thioredoxin-1 expression through TrkB/Akt/CREB pathway in SH-SY5Y cells. Biochimie 160, 55–60. 10.1016/j.biochi.2019.02.011 30796965

[B53] Braund T. A. Tillman G. Palmer D. M. Gordon E. Rush A. J. Harris A. W. (2021). Antidepressant side effects and their impact on treatment outcome in people with major depressive disorder: an iSPOT-D report. Transl. Psychiatry. 11 (1), 417. 34349116 10.1038/s41398-021-01533-1 PMC8338944

[B9] Burback L. Brult-Phillips S. Nijdam M. J. McFarlane A. Vermetten E. (2024). Treatment of posttraumatic stress disorder: a state-of-the-art review. Curr. Neuropharmacology 22 (4), 557–635. 10.2174/1570159X21666230428091433 PMC10845104 37132142

[B10] Calcia M. A. Bonsall D. R. Bloomfield P. S. Selvaraj S. Barichello T. Howes O. D. (2016). Stress and neuroinflammation: a systematic review of the effects of stress on microglia and the implications for mental illness. Psychopharmacology 233 (9), 1637–1650. 10.1007/s00213-016-4218-9 26847047 PMC4828495

[B11] Cheng Y. Tian D.-Y. Wang Y.-J. (2020). Peripheral clearance of brain-derived Aβ in alzheimer's disease: pathophysiology and therapeutic perspectives. Transl. Neurodegeneration 9, 1–11. 10.1186/s40035-020-00195-1 32381118 PMC7204069

[B12] Chou S. Davis C. Li M. (2021). Maternal immune activation and repeated maternal separation alter offspring conditioned avoidance response learning and antipsychotic response in male rats. Behav. Brain Research 403, 113145. 10.1016/j.bbr.2021.113145 33515643 PMC7902402

[B13] Cleal M. Fontana B. D. Parker M. O. (2021). The cognitive and behavioral effects of D-amphetamine and nicotine sensitization in adult zebrafish. Psychopharmacology 238 (8), 2191–2200. 10.1007/s00213-021-05844-5 33963883 PMC8292302

[B14] Correia A. S. Cardoso A. Vale N. (2023). Oxidative stress in depression: the link with the stress response, neuroinflammation, serotonin, neurogenesis and synaptic plasticity. Antioxidants 12 (2), 470. 10.3390/antiox12020470 36830028 PMC9951986

[B15] Cunha J. M. Masur J. (1978). Evaluation of psychotropic drugs with a modified open field test. Pharmacology 16 (5), 259–267. 10.1159/000136777 25444

[B16] Delgado P. L. (2000). Depression: the case for a monoamine deficiency. J. Clinical Psychiatry 61 (6), 7–11. 10775018

[B17] Deogracias R. Espliguero G. Iglesias T. Rodríguez-Peña A. (2004). Expression of the neurotrophin receptor trkB is regulated by the cAMP/CREB pathway in neurons. Mol. Cell. Neurosci. 26 (3), 470–480. 10.1016/j.mcn.2004.03.007 15234351

[B18] Ekundayo B. E. Obafemi T. O. Adewale O. B. Obafemi B. A. Oyinloye B. E. Ekundayo S. K. (2024). Oxidative stress, endoplasmic reticulum stress and apoptosis in the pathology of Alzheimer’s disease. Cell Biochemistry Biophysics 82 (2), 457–477. 10.1007/s12013-024-01248-2 38472715

[B19] Elfakharany S. A. Eskaros S. S. Azhary N. M. E. Abdelmonsif D. A. Zeitoun T. M. Ammar G. A. (2024). Neuroprotective role of selenium nanoparticles against behavioral, neurobiochemical and histological alterations in rats subjected to chronic restraint stress. Mol. Neurobiol. 61 (12), 10159–10181. 10.1007/s12035-024-04196-3 38703343 PMC11584447

[B20] Esmaeili Y. Yarjanli Z. Pakniya F. Bidram E. Łos M. J. Eshraghi M. (2022). Targeting autophagy, oxidative stress, and ER stress for neurodegenerative disease treatment. J. Control. Release 345, 147–175. 10.1016/j.jconrel.2022.03.001 35248646

[B21] Fabbri C. (2025). Treatment-resistant depression: role of genetic factors in the perspective of clinical stratification and treatment personalisation. Mol. Psychiatry 30 (5), 2210–2218. 10.1038/s41380-025-02899-0 39827221

[B22] Ferreira de Almeida T. L. Petarli G. B. Cattafesta M. Zandonade E. Bezerra O. M. d. P. A. Tristão K. G. (2021). Association of selenium intake and development of depression in Brazilian farmers. Front. Nutrition 8, 671377. 10.3389/fnut.2021.671377 34095192 PMC8173156

[B23] Gao Y. Wang C. Jiang D. An G. Jin F. Zhang J. (2022). New insights into the interplay between autophagy and oxidative and endoplasmic reticulum stress in neuronal cell death and survival. Front. Cell Dev. Biol. 10, 994037. 10.3389/fcell.2022.994037 36187470 PMC9524158

[B24] Habib M. Z. Ebeid M. A. El Faramawy Y. Saad S. S. El Magdoub H. M. Attia A. A. (2020). Effects of lithium on cytokine neuro-inflammatory mediators, Wnt/β-catenin signaling and microglial activation in the hippocampus of chronic mild stress-exposed rats. Toxicol. Applied Pharmacology 399, 115073. 10.1016/j.taap.2020.115073 32454056

[B25] Hamdan A. M. E. Alharthi F. H. J. Alanazi A. H. El-Emam S. Z. Zaghlool S. S. Metwally K. (2022). Neuroprotective effects of phytochemicals against aluminum chloride-induced Alzheimer’s disease through ApoE4/LRP1, wnt3/β-catenin/gsk3β, and TLR4/NLRP3 pathways with physical and mental activities in a rat model. Pharmaceuticals 15 (8), 1008. 10.3390/ph15081008 36015156 PMC9416484

[B26] Hassan W. Noreen H. Rehman S. Kamal M. A. da Rocha J. B. (2022). Association of oxidative stress with neurological disorders. Curr. Neuropharmacology 20 (6), 1046–1072. 10.2174/1570159x19666211111141246 34781871 PMC9886831

[B27] Herbert J. Lucassen P. J. (2016). Depression as a risk factor for Alzheimer’s disease: genes, steroids, cytokines and neurogenesis–what do we need to know? Front. Neuroendocrinology 41, 153–171. 10.1016/j.yfrne.2015.12.001 26746105

[B28] Hritcu L. Cioanca O. Hancianu M. (2012). Effects of lavender oil inhalation on improving scopolamine-induced spatial memory impairment in laboratory rats. Phytomedicine 19 (6), 529–534. 10.1016/j.phymed.2012.02.002 22402245

[B29] Hu S. Begum A. N. Jones M. R. Oh M. S. Beech W. K. Beech B. H. (2009). GSK3 inhibitors show benefits in an Alzheimer's disease (AD) model of neurodegeneration but adverse effects in control animals. Neurobiol. Disease 33 (2), 193–206. 10.1016/j.nbd.2008.10.007 19038340 PMC4313761

[B30] Ilic M. Ilic I. (2022). Worldwide suicide mortality trends (2000-2019): a joinpoint regression analysis. World Journal Psychiatry 12 (8), 1044–1060. 10.5498/wjp.v12.i8.1044 36158305 PMC9476842

[B31] Jiang Y. Zou D. Li Y. Gu S. Dong J. Ma X. (2022). Monoamine neurotransmitters control basic emotions and affect major depressive disorders. Pharmaceuticals 15 (10), 1203. 10.3390/ph15101203 36297314 PMC9611768

[B32] Li Y. Zhang J. Wan J. Liu A. Sun J. (2020). Melatonin regulates Aβ production/clearance balance and Aβ neurotoxicity: a potential therapeutic molecule for Alzheimer’s disease. Biomed. and Pharmacother. 132, 110887. 10.1016/j.biopha.2020.110887 33254429

[B33] Liang Y. Song P. Chen W. Xie X. Luo R. Su J. (2021). Inhibition of caspase-1 ameliorates ischemia-associated blood-brain barrier dysfunction and integrity by suppressing pyroptosis activation. Front. Cellular Neuroscience 14, 540669. 10.3389/fncel.2020.540669 33584203 PMC7874210

[B34] Liu Y. Zhao J. Guo W. (2018). Emotional roles of mono-aminergic neurotransmitters in major depressive disorder and anxiety disorders. Front. Psychology 9, 2201. 10.3389/fpsyg.2018.02201 30524332 PMC6262356

[B35] Liu D. Chen L. Zhao H. Vaziri N. D. Ma S.-C. Zhao Y.-Y. (2019). Small molecules from natural products targeting the Wnt/β-catenin pathway as a therapeutic strategy. Biomed. and Pharmacother. 117, 108990. 10.1016/j.biopha.2019.108990 31226638

[B36] Livak K. J. Schmittgen T. D. (2001). Analysis of relative gene expression data using real-time quantitative PCR and the 2− ΔΔCT method. Methods 25 (4), 402–408. 10.1006/meth.2001.1262 11846609

[B37] Magnuson B. Burdock G. Doull J. Kroes R. Marsh G. Pariza M. (2007). Aspartame: a safety evaluation based on current use levels, regulations, and toxicological and epidemiological studies. Crit. Reviews Toxicology 37 (8), 629–727. 10.1080/10408440701516184 17828671

[B38] Michaelson D. M. (2014). APOE ε4: the most prevalent yet understudied risk factor for Alzheimer's disease. Alzheimer's and Dementia 10 (6), 861–868. 10.1016/j.jalz.2014.06.015 25217293

[B39] Mohammadi N. Asle-Rousta M. Rahnema M. Amini R. (2021). Morin attenuates memory deficits in a rat model of alzheimer's disease by ameliorating oxidative stress and neuroinflammation. Eur. J. Pharmacol. 910, 174506. 10.1016/j.ejphar.2021.174506 34534533

[B40] Morris R. (1984). Developments of a water-maze procedure for studying spatial learning in the rat. J. Neuroscience Methods 11 (1), 47–60. 10.1016/0165-0270(84)90007-4 6471907

[B41] Mountaki C. Dafnis I. Panagopoulou E. A. Vasilakopoulou P. B. Karvelas M. Chiou A. (2021). Mechanistic insight into the capacity of natural polar phenolic compounds to abolish Alzheimer's disease-associated pathogenic effects of apoE4 forms. Free Radic. Biol. Med. 171, 284–301. 10.1016/j.freeradbiomed.2021.05.022 34019932

[B42] Pérez-Palma E. Andrade V. Caracci M. O. Bustos B. I. Villaman C. Medina M. A. (2016). Early transcriptional changes induced by Wnt/β‐Catenin signaling in hippocampal neurons. Neural Plast. 2016 (1), 4672841. 10.1155/2016/4672841 28116168 PMC5223035

[B43] Puglisi-Allegra S. Andolina D. (2015). Serotonin and stress coping. Behav. Brain Research 277, 58–67. 10.1016/j.bbr.2014.07.052 25108244

[B44] Ramez A. B. Islam I. H. El-Esawy R. Rania N. (2019). The prospective protective effect of selenium against chronic restraint stress-induced memory impairment in male albino rats. Med. J. Cairo Univ. 87 (June), 1563–1572. 10.21608/mjcu.2019.53576

[B45] Rayman M. P. (2012). Selenium and human health. Lancet 379 (9822), 1256–1268. 10.1016/S0140-6736(11)61452-9 22381456

[B46] Reinwald J. R. Becker R. Mallien A. S. Falfan-Melgoza C. Sack M. von Hohenberg C. C. (2018). Neural mechanisms of early-life social stress as a developmental risk factor for severe psychiatric disorders. Biol. Psychiatry 84 (2), 116–128. 10.1016/j.biopsych.2017.12.010 29397900

[B47] Sajjadi S. S. Foshati S. Haddadian-Khouzani S. Rouhani M. H. (2022). The role of selenium in depression: a systematic review and meta-analysis of human observational and interventional studies. Sci. Rep. 12 (1), 1045. 10.1038/s41598-022-05078-1 35058530 PMC8776795

[B48] Sari M. H. M. Ferreira L. M. Prado V. C. Nogueira C. W. Cruz L. (2022). Nano-based formulations as an approach for providing a novel identity for organoselenium compounds. Eur. J. Pharm. Biopharm. 178, 69–81. 10.1016/j.ejpb.2022.07.018 35932964

[B49] Shao X. Zhu G. (2020). Associations among monoamine neurotransmitter pathways, personality traits, and major depressive disorder. Front. Psychiatry 11, 381. 10.3389/fpsyt.2020.00381 32477180 PMC7237722

[B50] Shen M. Yang Y. Wu Y. Zhang B. Wu H. Wang L. (2019). L‐theanine ameliorate depressive‐like behavior in a chronic unpredictable mild stress rat model via modulating the monoamine levels in limbic–cortical–striatal–pallidal–thalamic‐circuit related brain regions. Phytotherapy Res. 33 (2), 412–421. 10.1002/ptr.6237 30474152

[B51] Sørensen J. Ø. Rasmussen A. Roesbjerg T. Verhulst F. C. Pagsberg A. K. (2022). Suicidality and self‐injury with selective serotonin reuptake inhibitors in youth: occurrence, predictors and timing. Acta Psychiatr. Scand. 145 (2), 209–222. 10.1111/acps.13360 34374070 PMC9292826

[B52] Taheri Zadeh Z. Rahmani S. Alidadi F. Joushi S. Esmaeilpour K. (2021). Depresssion, anxiety and other cognitive consequences of social isolation: drug and non‐drug treatments. Int. Journal Clinical Practice 75 (12), e14949. 10.1111/ijcp.14949 34614276

[B54] Varese F. Smeets F. Drukker M. Lieverse R. Lataster T. Viechtbauer W. (2012). Childhood adversities increase the risk of psychosis: a meta-analysis of patient-control, prospective-and cross-sectional cohort studies. Schizophr. Bulletin 38 (4), 661–671. 10.1093/schbul/sbs050 22461484 PMC3406538

[B55] Wang J. Um P. Dickerman B. A. Liu J. (2018). Zinc, magnesium, selenium and depression: a review of the evidence, potential mechanisms and implications. Nutrients 10 (5), 584. 10.3390/nu10050584 29747386 PMC5986464

[B56] Wang B.-J. Zheng W.-L. Feng N. Wang T. Zou H. Gu J.-H. (2018). The effects of autophagy and PI3K/AKT/m-TOR signaling pathway on the cell-cycle arrest of rats primary sertoli cells induced by zearalenone. Toxins 10 (10), 398. 10.3390/toxins10100398 30274213 PMC6215106

[B57] Yankelevitch-Yahav R. Franko M. Huly A. Doron R. (2015). The forced swim test as a model of depressive-like behavior. J. Visualized Experiments JoVE (97), 52587. 10.3791/52587 25867960 PMC4401172

[B58] Yavuz M. Dayanc E. D. Merve Antmen F. Keskinöz E. Altuntaş E. Dolu G. (2024). Relationships between trace elements and cognitive and depressive behaviors in sprague dawley and wistar albino rats. Front. Pharmacol. 15, 1367469. 10.3389/fphar.2024.1367469 38628647 PMC11018905

[B59] Zolfaghari F. Heydari Z. Zamani-Alavijeh F. Araban M. (2021). Determinants of postpartum depression according to the world health organization model: a review of literature. J. Health Syst. Res. 16 (4), 290–302.

